# The long-term effects of naprapathic manual therapy on back and neck pain - Results from a pragmatic randomized controlled trial

**DOI:** 10.1186/1471-2474-11-26

**Published:** 2010-02-05

**Authors:** Eva Skillgate, Tony Bohman, Lena W Holm, Eva Vingård, Lars Alfredsson

**Affiliations:** 1 Institute of Environmental Medicine, Karolinska Institutet, Box 210, SE-17177, Stockholm, Sweden; 2 Department of Medical Sciences, Occupational and Environmental Medicine, Uppsala University, Akademiska sjukhuset, SE-75185, Uppsala, Sweden; 3 Toronto Western Hospital Med West Building, 750 Dundas Street West, Box 36 Toronto, Ontario, M6J 3S3, Canada; 4 Skandinaviska Naprapathögskolan (Scandinavian College of Naprapathic Manual Medicine), Kräftriket 23A, SE-11419, Stockholm, Sweden

## Abstract

**Background:**

Back and neck pain are very common, disabling and recurrent disorders in the general population and the knowledge of long-term effect of treatments are sparse. The aim of this study was to compare the long-term effects (up to one year) of naprapathic manual therapy and evidence-based advice on staying active regarding non-specific back and/or neck pain. Naprapathy, a health profession mainly practiced in Sweden, Finland, Norway and in the USA, is characterized by a combination of manual musculoskeletal manipulations, aiming to decrease pain and disability in the neuromusculoskeletal system.

**Methods:**

Subjects with non-specific pain/disability in the back and/or neck lasting for at least two weeks (n = 409), recruited at public companies in Sweden, were included in this pragmatic randomized controlled trial. The two interventions compared were naprapathic manual therapy such as spinal manipulation/mobilization, massage and stretching, (*Index Group*), and advice to stay active and on how to cope with pain, provided by a physician (C*ontrol Group*). Pain intensity, disability and health status were measured by questionnaires.

**Results:**

89% completed the 26-week follow-up and 85% the 52-week follow-up. A higher proportion in the Index Group had a clinically important decrease in pain (risk difference (RD) = 21%, *95% CI: 10-30*) and disability (RD = 11%, *95% CI: 4-22*) at 26-week, as well as at 52-week follow-ups (pain: RD = 17%, *95% CI: 7-27 *and disability: RD = 17%, *95% CI: 5-28*). The differences between the groups in pain and disability considered over one year were statistically significant favoring naprapathy (p ≤ 0.005). There were also significant differences in improvement in bodily pain and social function (subscales of SF-36 health status) favoring the Index Group.

**Conclusions:**

Combined manual therapy, like naprapathy, is effective in the short and in the long term, and might be considered for patients with non-specific back and/or neck pain.

**Trial registration:**

Current Controlled Trials ISRCTN56954776.

## Background

Back and neck pain are very common, have multiple etiologies, and often recurs [1-4], and are among the most common reasons for seeking primary health care [5]. The existing literature suggests that there is little difference in effect between the available treatments [6].

One common non-invasive treatment is manual therapy, which is provided by several groups of health care providers such as naprapaths, physiotherapists, chiropractors and osteopaths. Manual therapy involves a variety of procedures directed at the neuromusculoskeletal structures.

The most well studied manual techniques for back and neck pain is spinal manipulation/mobilization and massage therapy, respectively. There is sufficient evidence from systematic reviews that spinal manipulation therapy (SMT) is an effective treatment for back pain [7-9]. Bronfort et al [8] concluded that the evidence supports SMT as an option for chronic low back pain in the most recent systematic review on SMT, using the method best evidence synthesis. Assendelft et al. found that SMT was as effective as other standard treatments for acute or chronic low back pain in a meta-analysis [7]. The SMT is recommended in evidence-based national guidelines for treatment of back pain in most countries [10]. A number of evidence-based guidelines for neck pain have recommended SMT, although a minority has not [11]. It is unclear if SMT have long-term effects [12].

Regarding massage therapy, the evidence from systematic reviews is strong that it is an effective treatment for low back pain [9,13]. It has, however, not been possible to draw any conclusions in evidence-based reviews on the effect of massage on neck pain because of contradictory results and a lack of studies of high quality [11,14].

There are also systematic reviews that include studies that in a pragmatic way have evaluated the effect of a combination of manual therapies. A review from 2008 concluded regarding neck pain without radicular symptoms, that manual therapy and exercise interventions are effective, but that none of the studied treatments are superior to any other in the short or long term [6].

In naprapathic manual therapy a combination of manual techniques (such as massage, muscle stretching, spinal manipulation and spinal mobilization) are used to increase physical function and decrease pain in the neuromusculoskeletal system. We have found few studies that have been set up to study the effect of such a pragmatic combination of several manual techniques on back and neck pain, and they have conflicting results [15-19].

Naprapathy is a health profession characterized by focusing on shortened or pathologic soft and connective tissues around the spine and other joints. Naprapathy is common in Sweden, Norway and Finland, and is also practiced in the USA where it was first initiated in 1907. Naprapaths have a five years full-time education, are part of the Swedish health and medical care system, and licensed from the National Board of Health and Welfare in Sweden. We have performed a pragmatic RCT with the aim of comparing the effect of naprapathic manual therapy (a combination of manual techniques) to the effect of evidence-based advice on staying active provided by a physician for non-specific back and/or neck pain. The intention was not to evaluate the different components in the treatments separately, but to compare the treatments, standardized as far as possible, the way they usually are carried out in outpatient clinics. The publication on the short-term effects showed significant differences between the groups regarding pain intensity, disability and perceived recovery, at 7- and 12-week follow-ups favoring naprapathy [20]. The aim of this study was to study the long-term effects (26 and 52 weeks respectively) of naprapathy for patients with non-specific back and/or neck pain, in the same trial.

## Methods

### Design Overview

This pragmatic randomized controlled trial, called "the Swedish BJORN-trial", was carried out in compliance with the Helsinki Declaration, and was approved by the Ethics Committee of the Karolinska Institutet (Diary No. 03-657).

### Setting and Participants

Participants were recruited by advertising mainly among employees (n = approximately 40,000) at two public companies in Stockholm, Sweden from March to September 2005. Potential participants were asked to contact the study administration if they fulfilled the inclusion criteria (pain now and the previous two weeks or longer in back and/or neck of the kind that brought about marked dysfunction at work and/or in leisure time). The study administrator made the first-step exclusions (symptoms too mild, pregnancy, specific diagnoses such as acute slipped disc or spinal stenosis, inability to understand Swedish, and recent visits to a manual therapist with the exception of massage). Subjects fulfilling the participation criteria were scheduled for an appointment at the study center where they gave their informed consent and answered an extensive self-administered questionnaire. Next an experienced physician (one of four) performed a medical examination, made a diagnosis, and prescribed medication if necessary. Further exclusions were made based on the following exclusion criteria: too mild symptoms (the physicians' *subjective *opinion based on the estimated pain and disability in the questionnaires filled in before the examination, and the results of the anamnesis and physical examination), evidence-based advice during the past month, surgery in the painful area, acute disc herniation, spondylolisthesis, stenosis or "red flags" [4].

Further details on design, material and methods are described elsewhere [20].

### Randomization and Interventions

Included subjects were assigned to two groups by randomization. An assistant not involved in the project prepared five hundred opaque, sequentially numbered sealed envelopes with cards numbered 1 or 2 (randomized by a computer), indicating the two interventions. Subjects were sequentially numbered in the order they came to the study center and received the assignment envelope with the corresponding number. The physician performed the unmasking after the assessments and the medical examination, so that the assistant, the physician and the patient were all blind to the group assignment until after all subjects' baseline data were collected.

The treatments in both groups were conformed to the patients' condition, but standardized as far as possible by several group meetings held in advance. The naprapaths were told only to use techniques they had learned at the education center. The content in the evidence-based advice and support were carefully discussed in group with the physicians in order to make the care reliable.

### Naprapathic Manual Therapy (Index Group)

For patients in the Index Group, one of eight participating experienced licensed naprapaths was contacted for a first appointment within a week. A maximum of six treatments were given within six weeks in the naprapaths private clinic, and a combination of naprapathic manual techniques (such as spinal manipulation/mobilization, massage and stretching) was given adapted to the patients' condition. Preventive and rehabilitating advice on for the patient applicable physical activity and ergonomics were often given. Each appointment lasted for about 45 minutes, and precise notes were kept about the treatment, the progress, and any adverse reactions.

### Evidence-Based Care Provided by a Physician (Control Group)

Evidence-based care is in this study defined as support and advice on staying active and on pain coping strategies, according to guidelines and evidence-based reviews [4,21,22]. This was given in direct conjunction with the medical examination (an additional 15 minutes) at the study center. The aim was to empower the patient with the understanding of the importance of staying active and living as normal a life as possible, including work and physical activities. The care also aimed to improve the pain coping strategies. Advice on exercises was general and adapted to the patient's condition. A booklet with examples of exercises and general information on back and neck pain was provided. Precise notes were kept and a second consultation was scheduled after three weeks. Additional consultation was offered if necessary.

### Outcomes and Follow-ups

All outcomes were self-rated by web-based (61%) or postal questionnaires five times during the year following the inclusion. The primary outcomes pain and disability were measured by the Chronic Pain Questionnaire (CPQ) with three items on pain and three on disability with a numerical 11-point scale [23-26]. In the current trial we changed the questions to concern the past four weeks instead of the past six months. A pain score was constructed from the mean of the three pain items and a disability score from the mean of the three disability items. Disability was also measured in a more detailed way by a modified version of the Whiplash Disability Questionnaire (WDQ), with 13 items, each with a numerical 11-point scale [27-29]. In the current context we modified the items by replacing the word "whiplash" with "back pain" or "neck pain". This disability score was the mean of the 13 items.

Four dichotomized outcomes were defined in advance based on what is believed to correspond to a clinically significant improvement [30-33]:

1) *improvement in pain: *at least a two-step decrease (compared to baseline) in pain score (CPQ).

2) *improvement in disability I: *at least a one-step decrease (compared to baseline) in disability score (CPQ).

3) *improvement in disability II: *at least a one-step decrease (compared to baseline) in disability score (WDQ).

4) *totally recovered*: a pain score less or equal to 1 and a disability score equal to 0 (CPQ).

Secondary outcome was health status measured with The Medical Outcomes Study Short Form-36 Health Survey (SF-36) after 26 and 52 weeks [34,35]. The secondary outcome "perceived recovery", reported on in the first publication based on the trial [20], was not measured at the long term follow-ups because of the risk of bias in recalling recovery back for time more than three months.

### Statistical Analysis

Power calculation based on the dichotomization of CPQ described above, determined the sample size to 400 patients and indicated a power of >80% to detect a relative risk of 1.2-1.3 for a clinically important improvement in pain or in disability.

All analyses were performed using an "intention to treat" principle [36]. To estimate the impact of missing responses, additional sensitivity analyses were performed, using multiple imputations with "predictive mean matching method" [37]. Changes in mean scores at follow-up compared to baseline and differences in changes between groups were calculated by t-test. To compare the groups regarding the dichotomized outcomes, relative risks (RR) and risk differences (RD) together with corresponding 95% confidence intervals (95% CI) were calculated. Baseline factors that differed between the treatment groups were considered with regard to their potential confounding effect by means of Mantel Haenszel's method [38]. None of the factors changed the result considerably (around 10% or more), so there was no need for adjusting for confounding from these factors [39].

Generalized Estimating Equations (GEE) were performed to analyze the effect on pain and disability (dichotomized) over the total follow-up time (7, 12, 26 and 52 weeks). The final model for improvement in pain and disability respectively, included the following terms in addition to the treatment variable: location of pain (back or neck), follow-up occasion and an interaction term between location of pain and follow-up occasions. The GEE method extends standard regression analysis, taking into account the covariance between repeated measurements of pain and disability [40,41].

In the analyses of the outcomes "improvement in pain" and "improvement in disability", patients with scores at baseline less than required to attain these improvements were excluded. In analyses of neck and back pain patients respectively, patients with concurrent pain in the neck and back (n = 25) were treated both as neck pain patients and back pain patients.

Crude SF-36 data were transformed and standardized using recommended procedures and to receive the dichotomization regarding good health, values from a Swedish population was used [34,35].

All analyses were performed by a statistician who was not involved in the project, using SAS statistical software version 9.1.3. An assistant not involved in the project handled all data registration.

## Results

**Figure 1:**
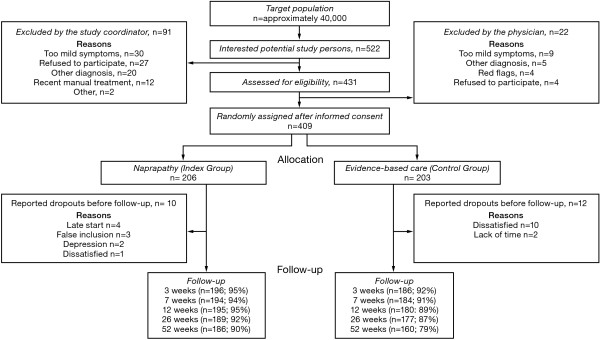
**Flow chart describing the progress of patients through the trial**.

Among the 522 subjects that contacted the trial administration, 431 were eligible and 409 were randomly assigned to one of the two treatments. The assigned had a mean age of 47 years, were mainly women (71%), and were mainly suffering from neck pain (58%). For many, duration of pain was more than a year (56%). Analyses of the treatments actually given in the Index Group showed that 98% received massage, 83% stretching, 57% spinal mobilization and 81% received spinal manipulation at the second consultation. Eighty-nine percent completed the 26- week follow-up and 85% the 52-week follow-up. The flow of participants through each stage of the trial and details about dropouts are shown in Figure 1.

Baseline demographics and clinical characteristics of the groups are shown in Table 1.

**Table 1:** Prognostic indicators

Prognostic indicators	Index Group (n = 206)	Control Group (n = 203)
Mean age (SD), years	46 (11)	48 (10)
Women, %	74	68
Location of the worst pain, %		
Neck	56	61
Back	36	34
Neck and back	8	5
Duration of pain, %		
<3 months	22	29
3-12 months	20	18
>12 months	58	54
Previous episodes of current pain in back and/or neck, %	88	85
Pain or trouble from five body regions or more, %	67	56*
Education, at least, %		
1-9 years	13	11
10-12 years	34	34
13-16 years	45	47
>16 years	8	8
Depression, %	22	24
Sleeping problems, %	26	29
Daily smoking, %	15	13
Physical training medium high or high effort at least 20 minutes each time, %		
Never	38	28*
1-2 times/week	21	33*
>3 times/week	41	39
Obesity, %	10	15
On sick leave now due to back/neck pain, %	3	5
Mean number of days absent from work the preceding 6 months (SD)	3 (17)	4 (20)
Physical demands at work, %	39	35
Job strain†, %	22	15
Bullying from superiors or workmates, %	13	14
Life events (> = 2) the preceding five years, %	81	75

*Statistical significant difference between groups (p ≤ 0.05).

† Swedish version of the demand-control-support model by Karasek-Theorell.

**Figure 2:**
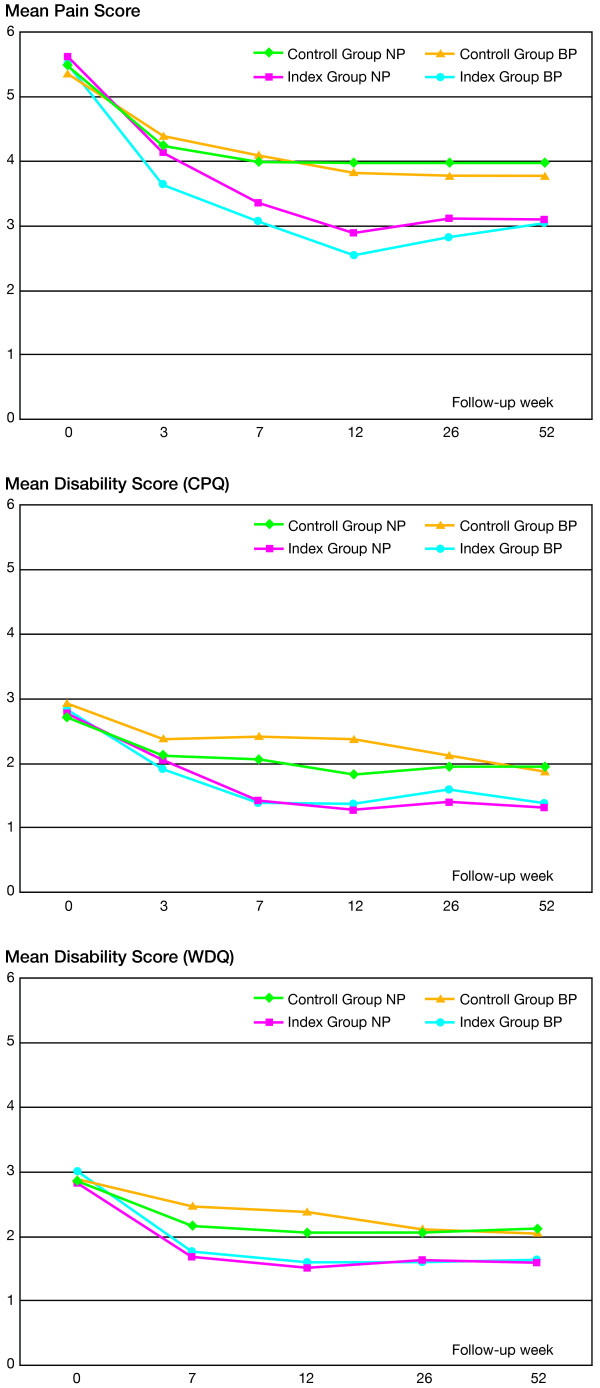
**The mean scores of pain and disability**. The mean scores of pain and disability for back pain patients (BP) and neck pain patients (NP) respectively during one year after inclusion.

Figure 2 shows the course of pain and disability for neck pain patients and back pain patients, respectively, over a year. Baseline values, changes in the mean of the outcomes for patients taking part in the follow-ups compared to baseline, and difference in mean changes between groups are shown in Table 2. There were statistically significant differences in changes in pain intensity and disability between the groups favoring the Index Group at 26 and 52 weeks.

**Table 2:** Differences in change in pain and disability between the groups

Baseline	26 weeks		52 weeks	
Baseline value	Change*	Diff. in change†	Change*	Diff. in change†
(95% CI)	(95% CI)	(95% CI) p-value	(95% CI)	(95% CI) p-value
**Pain **(CPQ)				
*Index group*				
5.5	2.6		2.5	
(5.3-5.8)	(2.3-2.9)		(2.2-2.9)	
n = 204	n = 188	1.0	n = 182	0.5
		(0.5-1.5)		(0.1-1.1)
		<0.001		0.021
*Control Group*				
5.4	1.6		2.0	
(5.2-5.7)	(1.3-2.0)		(1.6-2.3)	
n = 203	n = 176		n = 158	

**Disability **(CPQ)				
*Index group*				
2.7	1.4		1.5	
(2.5-3.0)	(1.0-1.7)		(1.2-1.8)	
n = 206	n = 186	0.6	n = 184	0.7
		(0.1-1.1)		(0.2-1.2)
		0.020		0.012
*Control group*				
2.8	0.8		0.8	
(2.3-3.1)	(0.4-1.2)		(0.4-1.2)	
n = 202	n = 176		n = 157	

**Disability **(WDQ)				
*Index group*				
3.0	1.3		1.4	
(2.8-3.2)	(1.1-1.6)		(1.2-1.7)	
n = 206	n = 189	0.5	n = 186	0.6
		(0.2-0.9)		(0.2-1.0)
		0.002		0.001
*Control group*				
3.0	0.8		0.8	
(2.8-3.3)	(0.6-1.1)		(0.5-1.1)	
n = 203	n = 178		n = 160	

Baseline values of pain and disability for the index and control groups, changes in the mean of the outcomes for patients taking part in the follow-up at 26 and 52 weeks, compared with baseline and differences in mean change between groups.

* The difference in the group mean of the outcomes at follow-up compared to baseline.

† The difference between the Index Group and Control Group with regard to change of group mean at follow-up.

A higher proportion in the Index Group had a clinically important improvement in pain intensity (RD = 21%, 95% CI: 10-30), disability on CPQ (RD = 11%, 95% CI: 4-22) and disability on WDQ (RD = 21%, 95% CI: 10-31) at 26 weeks, as well as at 52 weeks (pain: RD = 17%, 95% CI: 7-27, disability on CPQ: RD = 17%, 95% CI: 5-28 and disability on WDQ: RD = 19%, 95% CI: 8-30) (Table 3). A higher proportion in the Index Group was totally recovered at 26 weeks (RD = 11%, 95% CI: 4-19) and at 52 weeks (RD = 7%, 95% CI: (-1)-15) (Not in table). Generalized Estimating Equations (GEE) analyses showed that differences between the groups considered over one year were statistically significant regarding an improvement in pain (p = 0.002), and disability on CPQ (p = 0.005) and disability on WDQ (p < 0.001), favoring the Index Group. Sensitivity analyses performed to evaluate potential bias from loss of follow-up showed no systematic differences in results between analyses with and without imputed primary outcome values.

**Table 3:** Proportion of clinical significant improvements, the relative risk and the risk difference

Improvement	Index Group	Control Group	RR	RD	p-value
	(imp/not imp)†	(imp/not imp)†	(95% CI)	(95% CI)	
***26 weeks***					
Pain*	65%	44%	1.5	21%	
	(n = 120/65)	(n = 78/99)	(1.2-1.8)	(10-30)	<0.001
Disability*	74%	63%	1.2	11%	
(CPQ)	(n = 110/38)	(n = 82/48)	(1.0-1.4)	(4-22)	0.043
Disability*	66%	45%	1.4	21%	
(WDQ)	(n = 114/59)	(n = 75/90)	(1.2-1.8)	(10-31)	<0.001

***52 weeks***					
Pain*	67%	50%	1.3	17%	
	(n = 120/59)	(n = 79/79)	(1.1-1.6)	(7-27)	0.002
Disability*	75%	58%	1.3	17%	
(CPQ)	(n = 109/37)	(n = 68/49)	(1.1-1.5)	(5-28)	0.005
Disability*	68%	49%	1.4	19%	
(WDQ)	(n = 116/55)	(n = 72/75)	(1.1-1.7)	(8-30)	<0.001

The proportion of patients that reached clinically significant improvements in the intervention groups, the relative risk (RR), and the risk difference (RD), with corresponding 95% confidence intervals (95% CI) and p-values at 26 and 52 weeks follow-ups.

* A clinically important decrease corresponding to at least a two-step decrease in pain score from baseline, or at least a one-step decrease in disability score from baseline, respectively.

† Numbers of very much improved/not very much improved in the intervention groups.

Health related quality of life (SF-36) were better in the Index Group at 26 weeks and at 52 weeks follow-ups, but the differences were only statistically significant regarding the dimensions bodily pain and social function (Table 4).

**Table 4:** Health related quality of life (SF-36), the relative risk and the risk difference

Good quality of life	Index Group	Control Group	RR	RD	p-value
(Subscales of SF-36)	proportion (good/not good)*	proportion (good/not good)*	(95% CI)	(95% CI)	
***Base line***					
**Bodily Pain**	2%	3%	-	-	-
	(n = 4/202)	(n = 6/197)			
**Physical Function**	17%	22%	-	-	-
	(n = 34/166)	(n = 45/156)			
**Social Function**	35%	33%	-	-	-
	(n = 72/132)	(n = 66/137)			
**Role Emotion**	55%	57%	-	-	-
	(n = 114/92)	(n = 116/87)			
**General Health**	34%	35%	-	-	-
	(n = 69/133)	(n = 70/129)			

***26 weeks***					
**Bodily Pain**	37%	24%	1.2	13%	
	(n = 69/120)	(n = 43/133)	(1.0-1.4)	(3-21)	0.012
**Physical Function**	41%	35%	1.1	6%	
	(n = 75/110)	(n = 61/113)	(0.9-1.3)	((-5)-16)	0.285
**Social Function**	51%	37%	1.3	14%	
	(n = 95/91)	(n = 65/110)	(1.1-1.5)	(4-24)	0.008
**Role Emotion**	57%	48%	1.2	9%	
	(n = 106/79)	(n = 83/91)	(1.0-1.5)	((-1)-20)	0.069
**General Health**	38%	36%	1.0	2%	
	(n = 70/116)	(n = 63/112)	(0.9-1.2)	((-8)-12)	0.748

***52 weeks***					
**Bodily Pain**	39%	28%	1.2	11%	
	(n = 71/113)	(n = 45/114)	(1.0-1.4)	(0-20)	0.045
**Physical Function**	42%	32%	1.2	10%	
	(n = 76/105)	(n = 50/107)	(1.0-1.4)	(0-20)	0.055
**Social Function**	49%	36%	1.2	13%	
	(n = 89/92)	(n = 58/101)	(1.0-1.5)	(2-23)	0.018
**Role Emotion**	55%	46%	1.2	9%	
	(n = 101/84)	(n = 72/85)	(1.0-1.5)	((-2)-19)	0.107
**General Health**	39%	34%	1.1	5%	
	(n = 70/110)	(n = 54/106)	(0.9-1.3)	((-5)-15)	0.326

The proportion of patients with good health related quality of life (subscales of SF-36) in the intervention groups at baseline and at follow-ups, the relative risk (RR), and the risk difference (RD), with corresponding 95% confidence intervals (95% CI) and p-values at follow-ups.

* Numbers of persons with good/not good subscales of health related quality of life (SF-36) in the intervention groups.

Additional analysis on back and neck pain patients separately are presented in Table 5. Differences in change in pain intensity and disability were statistically significant for neck pain patients favoring the Index group at 52 weeks follow-up. The differences in changes were also higher in the Index group for back pain patients at 52 weeks, but only the changes in disability (WDQ) were statistically significant.

**Table 5:** Differences in change in pain and disability between the groups. Neck and back pain separately

Baseline	26 weeks		52 weeks	
Baseline value	Change*	Diff. in change†	Change*	Diff. in change†
(95% CI)	(95% CI)	(95% CI)	(95% CI)	(95% CI)
		p-value		p-value
**Neck pain **(CPQ)				
*Index group*				
5.6	2.5		2.5	
(5.4-5.9)	(2.1-2.9)		(2.0-3.0)	
n = 129	n = 117	0.9	n = 112	0.8
		(0.3-1.6)		(0.2-1.4)
		0.004		0.012
*Control group*				
5.5	1.6		1.7	
(5.2-5.8)	(1.1-2.0)		(1.3-2.1)	
n = 134	n = 115		n = 101	
**Back pain **(CPQ)				
*Index group*				
5.5	2.7		2.4	
(5.2-5.8)	(2.3-3.1)		(2.0-3.0)	
n = 92	n = 86	1.1	n = 83	0.1
		(0.4-1.7)		((-0.6)-0.8)
		0.003		0.712
*Control group*				
5.4	1.6		2.3	
(5.5-5.7)	(1.1-2.2)		(1.8-2.9)	
n = 80	n = 70		n = 65	

**Neck disability **(CPQ)				
*Index group*				
2.8	1.3		1.4	
(2.4-3.1)	(1.0-1.8)		(1.1-1.8)	
n = 131	n = 115	0.6	n = 116	0.7
		(0.0-1.3)		(0.1-1.4)
		0.044		0.023
*Control group*				
2.7	0.7		0.7	
(2.3-3.1)	(0.2-1.2)		(0.2-1.2)	
n = 133	n = 114		n = 100	
**Back disability **(CPQ)				
*Index group*				
2.8	1.2		1.5	
(2.4-3.3)	(0.7-1.8)		(0.9-2.0)	
n = 92	n = 86	0.5	n = 82	0.5
		((-0.3)-1.4)		((-0.4)-1.4)
		0.198		0.282
*Control group*				
3.0	0.7		1.0	
(2.4-3.5)	(0.1-1.3)		(0.3-1.6)	
n = 80	n = 71		n = 65	

**Neck disability **(WDQ)				
*Index group*				
3.0	1.2		1.3	
(2.7-3.3)	(0.9-1.5)		(1.0-1.6)	
n = 131	n = 118	0.4	n = 115	0.5
		((-0.1)-0.8)		(0.1-1.0)
		0.087		0.016
*Control group*				
3.0	0.8		0.8	
(2.7-3.3)	(0.5-1.2)		(0.4-1.1)	
n = 134	n = 116		n = 104	
**Back disability **(WDQ)				
*Index group*				
3.2	1.4		1.5	
(2.8-3.5)	(1.1-1.9)		(1.1-1.9)	
n = 92	n = 86	0.7	n = 84	0.6
		(0.2-1.3)		(0.1-1.2)
		0.006		0.032
*Control group*				
3.0	0.7		0.9	
(2.6-3.4)	(0.4-1.1)		(0.5-1.3)	
n = 80	n = 71		n = 66	

Baseline values of pain and disability for the neck and back pain patients respectively in index and control groups, changes in the mean of the outcomes for patients taking part in the follow-up at 26 and 52 weeks, compared to baseline and differences in mean change between groups.

* The difference in the group mean of the outcomes at follow-up compared to baseline.

† The difference between the Index Group and Control Group with regard to change of group mean at follow-up.

## Discussion

This pragmatic RCT was performed with the aim of investigating if naprapathic manual therapy is effective for non-specific back and/or neck pain. The control treatment was support and advice to stay active and on pain coping strategies [4,21,22]. We previously reported that naprapathic manual therapy was statistically and clinically significant more effective than the control treatment in the short term (up to 12 weeks) regarding pain intensity, disability and perceived recovery [20]. The findings of the present study were that the clinically and statistically significant differences in pain intensity and disability between the groups remained at 26 and 52 weeks, and that the differences between the groups considered over one year were statistically significant (p < 0.01) also when consideration was taken to the covariance between the repeated measurements. These results are unique since the long-term effects of naprapathic manual therapy have never been scientifically evaluated before.

Separate subgroup analysis of back and neck pain patients showed similar results, but indicated that the naprapathic manual treatment might be more effective for neck pain patients. A limitation in this discussion is that the back pain subgroup is small and the statistical power low.

The non-specific effects of the hands-on approach and the potentially intensive patient-therapist interaction in the Index Group due to more treatment sessions have probably contributed to the results. We do not believe that this difference in attention is the only reason to the results. Specific effects from the manual therapy on the function in the neuromusculoskeletal system probably also contributed. This assumption is made based on the documented effect of two of the manual techniques in naprapathic manual therapy, spinal manipulation/mobilization and massage, respectively [7-9,12,13,42]. The combined manual techniques delivered in naprapathy might have enabled patients to be more physically active and further to be able to practice a more internal locus of control [43] regarding the management of the back and neck pain problems. At the 26 weeks follow up 11% in the Index group and 6% in the Control Group had taken additional naprapathic manual therapy the preceding six months. At 52 weeks the proportion was 14% in the Index group and 4% in the Control Group. Thus the long-term effect in this trial is probably not due to additional naprapathic manual therapy in the Index Group.

No study is published that has evaluated the long-term effect of naprapathic manual therapy, which precludes comparing the results to earlier findings. Some trials have evaluated the long-term effect of other manual therapy strategies where several manual techniques as spinal manipulation/mobilization and soft tissue techniques as massage are combined, as is the case in naprapathic manual therapy. The UK BEAM pragmatic trial estimated the effect of adding exercise classes, a spinal manipulation package (a combination of several manual techniques), or spinal manipulation followed by exercise, to "best care" in general practice for patients consulting with back pain [15]. Relative to "best care," the spinal manipulation package with or without exercise achieved only a small benefit at the 12-month follow-up. Dziedzic et al. evaluated the effect of a combination of manual techniques on neck pain. No additional effect was recorded after six months when adding such manual therapy to advice and exercises [16]. Hoving et al. compared a manual therapy strategy (combination of manual techniques and coordination or stabilization techniques), physical therapy strategy and continued care by the general practitioner for neck pain patients [17]. Manual therapy strategy speeded up recovery in the short term, but the differences between the treatments groups at the 12-month follow-up were small. In two relatively small trials, combined manual therapies were more effective for neck pain than a minimal intervention approach [19], but only marginally more effective for low back pain than advice to stay active [18]. In summary, our trial recommends a more obvious benefit from combined manual therapy in the long term, than the summarized results in earlier published trials.

Strengths of our trial include the great number of subjects included and the relatively few dropouts, which led to a high internal validity. The kind of back and neck pain studied is very frequent, enabling a generalization of the results to a large proportion of the population. Another strength is that the results are presented with consideration taken not only to statistical significance, but also to clinically important changes in pain and disability [33].

The trial was not designed to evaluate the different components in the compared treatments, but to compare a treatment with unknown effect (naprapathic manual therapy) to a treatment with a well known positive effect (advice and support on staying active) on back and neck pain. Accordingly we cannot tell what in the naprapathic manual therapy that has a positive effect. Instead, the design enables a generalization of the results to outpatient clinics, something that we consider a strength. The treatment strategies differed regarding the number of treatments offered and the hands-on approach, which partly might explain the differences in outcomes between the groups. Nevertheless, another extensive trial of manual therapy for back pain had the same control treatment as in this trial. They found only small benefits from manual therapy. The choice of control treatment was in this case discussed as an explanation for the smaller differences between the groups than expected [15].

We did not measure the subjects' expectations of the effect of the interventions before the trial started. A detailed explanation of the control intervention would have been like exposing all to this intervention. Before inclusion in the study, all potential study persons were asked why they considered participating. Sixty percent of the assigned wanted to see a naprapath, which may indicate an expectation bias.

Typically, the most obvious effect of manual therapy is seen in the short term [15,17,19]. In the long term there are seldom clinically important differences between the treatments compared. This lack of long-term effects might be interpreted as if manual therapy is not an important alternative for back and neck pain. However, a short-term effect without a long-term effect might still be meaningful from the patient's point of view. To presume that patients are interested in receiving treatments that offers a quick diminishing of symptoms is probably not too provocative, especially when back and neck pain very often are recurrent. The long-term effects of treatments are of more interest from a public health perspective. When the society is to decide what treatment alternatives should be part of the publicly financed health care, the long-term effects are highly relevant, especially if cost effectiveness is taken into consideration. Distinguishing between the patient's health perspective and the community's public health perspective when reporting the results might contribute to a broader perspective in future studies.

We plan to report on the more public health oriented outcomes as cost utility and sick leave as observed in the present trial, when such data have been analyzed.

## Conclusions

Compared to evidence-based care provided by a physician, naprapathic manual therapy implied a greater improvement in pain and disability for patients with non-specific back and/or neck pain in the short as well as in the long term. The trial adds to the knowledge that recommending a combination of manual therapies, as naprapathic manual therapy, may be an alternative to consider in primary health care for these patients. Since back and neck pain are among the most common reasons for seeking primary health care our results may be of broad interest and importance.

## Competing interests

The sources of funding had no influence on the design, the conduct or the reporting of the trial. The authors declare that they have no competing interests. ES is a part time employee at the Scandinavian College of Naprapathic Manual Medicine and a scientific advisor to the Swedish Naprapathic Association. LA is a scientific advisor to the Scandinavian College of Naprapathic Manual Medicine. These potential conflicting interests has not influenced the interpretation of data or presentation of information in studies based on the trial.

## Authors' contributions

The authors ES, EV and LA have made substantial contributions to conception and design of the trial, acquisition and analysis of data. ES and TB are the main authors and EV, LA and LH have all been involved in interpretation of data, drafting the manuscript and revising it critically. All authors read and approved the final manuscript.

## Pre-publication history

The pre-publication history for this paper can be accessed here:

http://www.biomedcentral.com/1471-2474/11/26/prepub
